# S1 guidelines of the German Society of Neurology for Viral Meningoencephalitis- AWMF Registration No.: 030–100

**DOI:** 10.1186/s42466-026-00487-3

**Published:** 2026-04-17

**Authors:** Uta Meyding-Lamadé, Eva Maria Craemer, Kemal Aydin, Christian Jacobi, Judith Wagner, Bettina Pfausler, Hilmar Prange, Volker Schuchardt, Solveig Mosthaf, Matthias Mehling, Matthias Klein, Burc Bassa

**Affiliations:** 1 Krankenhaus Nordwest GmbH, Department of Neurology, Klinik für Neurologie, Steinbacher Hohl 2-26, 60488 Frankfurt, Germany; 2 Heraeus Medical GmbH, Wehrheim, Germany; 3 Sankt Katharinen Krankenhaus, Department of Neurology, Frankfurt, Germany; 4 Evangelisches Klinikum, Department of Neurology, Gelsenkirchen, Germany; 5 Universitätsklinik Innsbruck, Department of Neurology, Innsbruck, Austria; 6 Universitätsklinik Göttingen, Department of Neurology, Göttingen, Germany; 7 Klinik Lahr, Department of Neurology, Lahr, Germany; 8 Universitätsklinikum Schlesweig-Holstein, Germany {representive of the young neurologists, Deutsche Gesellschaft für Neurologie (DGN)}, Kiel and Lübeck, Germany; 9 Universitätsspital Basel, Basel, Switzerland; 10 LMU Klinikum, Department of Neurology, Munich, Germany

## Abstract

The guidelines ´Viral meningoencephalitis´ emphasize early and aggressive management, including rapid diagnosis through cerebrospinal fluid (CSF) analysis and neuroimaging (MRI).Treatment focuses on supportive care, with specific antiviral therapy (like acyclovir for herpes encephalitis) available for certain viral causes. These Guidelines also address the importance of prompt antibiotic treatment for bacterial meningitis when suspected, and the use of dexamethasone alongside antibiotics in some cases.

## **What’s new?**

- A Danish observational study analyzed 1066 cases of viral meningitis. On admission, headache was one of the most common symptoms (95% of cases), followed by history of fever or presence of fever (71%), hyperacusis or photophobia (67%) and neck stiffness (36%). Only 28% had the typical triad of headache, neck stiffness and hyperacusis or photophobia. Enteroviruses (EV) were detected in 419 (39%) cases, HSV-2 in 171 (16%) cases and VZV in 162 (15%) cases. In 31 (3%) cases different viruses were detected simultaneously and in 283 (27%) cases no pathogen could be identified. In summary, the outcome of viral meningitis was similar in patients with different etiologies, including those with suspected viral meningitis but no identified pathogen [1].
- In May 2023, an increased number of cases of echovirus-11 (E11) in newborns have been reported in France, 7 newborns died due to the infection. Following the announcement by the WHO, other EU member states (England, Croatia, Spain, Sweden, Northern Ireland) have reported cases of E-11. Echoviruses belong to the group of enteroviruses and can cause severe clinical pictures such as meningitis, sepsis, myocarditis or fulminant liver failure in newborns [2].
- Since May 2022, there has been an increase in cases of human monkeypox (MPXV) outside Africa; in July 2022, hMPXV was declared a global health threat by the WHO. In Germany alone, 3671 cases have been reported to the RKI in 2022 and 16 cases in 2023 (as of 08/2023). Transmission in the current outbreak is predominantly sexual, although other transmission routes are possible. Vaccinations have been offered to men with frequently changing sexual partners since July 2022.
- The range of drugs for viral diseases has been expanded in recent years. These include the neuraminidase inhibitors with efficacy against orthomyxoviruses (zanamivir (inhaled), oseltamivir (oral) and peramivir (intravenous)), the broad-spectrum anti-herpetics adefovir (also effective against HIV and HBV) and lobucavir. However, the efficacy of any of these preparations in viral meningoencephalitis has not been evaluated in controlled studies.
- For the treatment of CNS infections caused by influenza viruses, only neuraminidase inhibitors are currently available as clinically effective medications. The neuraminidase inhibitors oseltamivir and zanamivir block the activity of the viral neuraminidase and thus the release of newly formed viruses. They are effective against both influenza A and influenza B viruses. Resistance to neuraminidase inhibitors remains uncommon, is subtype-dependent, and usually emerges in cases of inadequate dosing or prolonged treatment duration. The susceptibility of the circulating virus strains is monitored as part of national and international surveillance systems.
- PCR panel diagnostics and next-generation sequencing (NGS) are increasingly being integrated into routine clinical practice for the evaluation of CNS infections.
- Serum procalcitonin measurement is a useful adjunct in distinguishing bacterial from viral meningoencephalitis, as elevated levels are generally indicative of bacterial infection. In addition, cerebrospinal fluid (CSF) lactate concentrations provide valuable discriminatory power between bacterial and viral etiologies. Both parameters can be assessed using point-of-care testing. Definitive pathogen identification is typically achieved through polymerase chain reaction (PCR) analysis of CSF obtained via lumbar puncture.
- Autoimmune encephalitis must also be considered in the differential diagnosis—particularly when no pathogen or relevant antibodies (e.g., anti-NMDA receptor, CASPR2, LGI1) can be identified—which presents a significant diagnostic challenge [3, 4].
- Moreover, autoimmune encephalitis may be triggered by a preceding viral encephalitis. This has been well documented in the context of HSV-1 encephalitis, which can lead to the development of anti-NMDA receptor antibody-positive autoimmune encephalitis [5].
- Emerging and atypical viral pathogens causing meningoencephalitis are increasingly detected in Western countries. These include dengue virus (Flaviviridae), Nipah virus (Paramyxoviridae), West Nile virus and Japanese encephalitis virus (both Flavivirus genus), and Toscana virus (Phlebovirus genus).
- Rabies is still the tenth most common infectious disease worldwide, with an estimated 60,000 deaths per year, although the number of unreported cases in Africa and Asia is estimated to be high [6]. Rabies is endemic in many tropical countries. There is also a worldwide risk of infection through contact with bats and flying foxes. Two inactivated vaccines are available in Germany.
- ‘Polio-like’ syndromes are most commonly associated with enteroviruses, particularly EV-D68, EV-A71, and EV-B88. In contrast, infections with Japanese encephalitis virus, tick-borne encephalitis (TBE) virus, parechoviruses, SARS-CoV-2, or Zika virus may rarely result in similar clinical presentations.
- Ebola virus disease (EVD) cases were reported in the Democratic Republic of Congo in 2021 and 2022. Due to the high case fatality rate, larger outbreaks are typically self-limiting. The most recent outbreak, occurring between September 2022 and January 2023, was caused by the Sudan Ebola virus and resulted in over 160 confirmed cases and 77 deaths in the western and central regions of Uganda.

## The most important recommendations at a glance

- The diagnosis of suspected viral encephalitis should be based on the clinical history, neurological examination, cerebrospinal fluid (CSF) analysis, and, where possible, detection of the causative pathogen in the CSF.
- The diagnostic work-up of CNS infections should include serological and molecular techniques, such as PCR and multiplex panel diagnostics from cerebrospinal fluid. In cases of suspected encephalitic syndromes, cranial MRI and EEG are indicated. Cranial CT (CCT) is inadequate for early diagnosis and should be reserved for initial screening or to exclude contraindications to lumbar puncture.
- In patients presenting with encephalitic symptoms and clinical suspicion of herpes simplex virus (HSV) encephalitis, intravenous acyclovir therapy must be initiated without delay. If lumbar puncture is contraindicated—e.g. due to signs of raised intracranial pressure—empirical acyclovir treatment should be commenced based solely on clinical suspicion, without awaiting cerebrospinal fluid (CSF) diagnostics.
- Acyclovir is also indicated for the treatment of varicella zoster virus (VZV) encephalitis. For cytomegalovirus (CMV) encephalitis, ganciclovir or foscarnet may be used. The use of corticosteroids as adjunctive therapy remains unproven and is not routinely recommended.
- In the absence of pathogen detection, autoimmune encephalitis—such as forms associated with antibodies against NMDA receptors or other neuronal targets—should be considered in the differential diagnosis.
- Patients with acute viral encephalitis should receive monitoring and care in an intensive care setting.
- Uncomplicated viral meningitis should be managed symptomatically with antipyretics and analgesics.

## Introduction

Viral encephalitis is a potentially debilitating neurological disease that is associated with significant morbidity during the acute illness and often complicated by persistent neurological deficits. Historically, an etiologic agent has been identified in only approximately 50% of encephalitis cases. Recent advances in molecular testing and the identification of a rapidly growing number of autoimmune diseases have significantly influenced the evaluation and treatment of encephalitis. In immunologically competent patients, viral encephalitis in temperate latitudes is mainly caused by a small group of viruses: Herpes simplex virus type 1 (HSV-1), varicella-zoster virus (VZV), Epstein-Barr virus (EBV), mumps, measles and enteroviruses (EV). HSV-1 and Enteroviruses are also common in children; VZV encephalitis has become rare after general pediatric vaccination. In children and young adults, autoimmune diseases such as anti-N-methyl-D- aspartate receptor (NMDAR) antibody encephalitis account for about 20–30% of cases, although they can occur at any age.

Prognosis is largely determined by the underlying pathogen, as well as the patient’s general health status and age. In the absence of treatment, herpes simplex virus encephalitis (HSVE) is associated with a mortality rate of up to 70%. Antiviral therapy reduces mortality to approximately 20–30%. A retrospective cohort study from France examined mortality and hospital readmission rates among all patients diagnosed with HSVE between 2015 and 2022.

Within six months of the initial HSVE, 10.1% of cases were readmitted. Overall, 16.5% of all patients died; patients requiring intensive care died in 20.8% of cases [7]. 

Some viral diseases that are rare in Europe, such as rabies, West Nile encephalitis (WNE) and Japanese encephalitis B (JEV), have a high mortality rate in the absence of specific treatment [8–11]. Cases of viral diseases such as the Zika virus (ZIKV) are also increasingly occurring in Europe due to migration and travel abroad.

Virustatics (acyclovir) are available for viruses of the Herpesviridae family, while supportive treatment is required for other viral diseases such as EV or WNV for which no effective antiviral therapy is available.

In addition to direct pathogen-induced damage, secondary immunologically mediated complications may arise, such as vasculitis and autoimmune encephalitis.

Anti-NMDA receptor (NMDAR) antibody encephalitis—the most common form of antibody-associated encephalitis—can be triggered by various infections, HSVE. Secondary autoimmune encephalitis typically develops weeks to months after the initial infection and should be considered in patients who exhibit new or worsening neurological symptoms despite appropriate aciclovir therapy. If autoimmune encephalitis is confirmed or strongly suspected, immunotherapy is indicated.

Acute transverse myelitis (ATM) is typically immune-mediated rather than caused by direct pathogen invasion.

In particular, infections with influenza viruses, mycoplasmas, and adenoviruses are thought to be predominantly of post- or parainfectious origin.

Patients with encephalitis of unknown etiology pose a particular diagnostic challenge. The diagnostic workup can be refined through additional investigations, such as follow-up brain MRI, repeated lumbar punctures, and tumor screening in cases suggestive of a paraneoplastic syndrome. In suspected antibody-negative autoimmune encephalitis, empirical immunomodulatory therapy may be warranted. Next-generation sequencing (NGS) of cerebrospinal fluid, blood, or other relevant samples should be considered to detect novel or unexpected pathogens. If autoimmune encephalitis is suspected but standard antibody panels are negative, immunohistochemical testing in a specialized reference laboratory is recommended. In cases of focal lesions on MRI—especially those with contrast enhancement or in immunocompromised patients—a brain biopsy may be necessary to establish a definitive diagnosis.

Advances in the understanding of autoimmune encephalitis, along with the development of molecular diagnostics such as next-generation sequencing (NGS), have significantly improved diagnostic accuracy and patient outcomes. However, effective antiviral therapies remain limited for non-herpes viral pathogens.

Distinguishing between immune-mediated encephalopathy or encephalitis and direct pathogen-mediated encephalitis can be challenging. Growing evidence suggests that a number of pathogens may initiate immune responses that lead to the production of autoantibodies as part of the infectious process. This has been demonstrated, for example, in cases of anti-NMDA receptor encephalitis following HSVE. In general, timely diagnosis and early initiation of appropriate treatment—when available—are crucial for reducing both morbidity and mortality [9, 11, 12]. 

Advances in neuroimaging, particularly magnetic resonance imaging (MRI), and molecular diagnostic techniques such as polymerase chain reaction (PCR) have significantly improved the identification of viral central nervous system (CNS) infections and facilitated the implementation of targeted therapeutic strategies. The introduction of selective neuraminidase inhibitors has expanded treatment options for infections caused by orthomyxoviruses.

Intravenous peramivir is not approved for routine use in Germany and can only be sourced through pharmacies abroad.

## Classification of Clinical Presentations

Viral meningitis typically presents with headache, nausea, occasional vomiting, neck stiffness, and photophobia or phonophobia. Focal neurological deficits and impaired consciousness are not characteristic features. Cerebrospinal fluid (CSF) analysis usually shows a moderate pleocytosis (typically < 1000 cells/µL), with only mild or no elevation in protein and lactate levels. Symptoms generally resolve spontaneously within several days to a few weeks, even in the absence of specific antiviral therapy.

**Acute viral (meningo)encephalitis** is defined by altered mental status—both qualitative and quantitative disturbances of consciousness—and is often, though not invariably, accompanied by focal neurological deficits such as paresis, aphasia, or focal/generalized seizures. Meningeal signs may be absent. A preceding systemic illness (e.g., rubella, measles, mumps, varicella, exanthema subitum) or a prodromal phase (e.g., in herpes simplex virus encephalitis, tick-borne encephalitis, or enteroviral infections including poliomyeloencephalitis) is common. In some cases, symptoms of the primary infection may have occurred days to weeks earlier.

An overview of the most relevant viral pathogens causing meningitis and meningoencephalitis in Europe is provided in Tables 1 and 2 shows the diagnostic criteria of viral encephalits.

**Table 1:** Acute and subacute neurological syndromes caused by viral pathogens (the pathogens that are particularly relevant in Central Europe are in bold) [13]

Syndrome	Clinical Symptoms	Possible Pathogens
Aseptic meningitis	Headache, neck pain, fever, meningismus, photophobia, phonophobia, fatigue, myalgia	**Coxsackievirus**, Echovirus, Adenovirus, **HSV-2**, VZV, Phlebovirus (Toscana fever/Italy), Poliovirus, Measles virus, TBEV, **Mumps virus**, EBV, Rubella virus, Enteroviruses (e.g., Enterovirus 71), **HIV**, Parvovirus B19, HHV-6, Dengue virus
Meningoencephalitis	Symptoms similar to aseptic meningitis; additionally: impaired consciousness, delirium, seizures, aphasia, apraxia, hemiparesis, cognitive impairment. Complications: status epilepticus, cerebral edema, extrapyramidal/movement disorders	**HSV**, **VZV**, Adenovirus, **TBEV**, Measles virus, CMV, Rabies virus, **Enteroviruses** (e.g., Enterovirus 71), Vaccinia virus, **HIV**, Lassa virus, Japanese encephalitis virus (JEV), West Nile virus, Poliovirus, Hantaviruses
Encephalopathy	Chronic: dementia. (Sub)acute: headaches, psychiatric symptoms, impaired consciousness	**HIV**, **Polyomaviruses (JCV**), Yellow fever virus, Hepatitis C virus, Lassa virus
Hemorrhagic fever with CNS involvement	Fever, headache, abdominal pain, muscle pain, vomiting, diarrhea, shock, renal failure, meningismus, seizures, impaired consciousness, coagulopathy	**Hantaviruses** (e.g., Hantaan, Puumala), Filoviruses (Marburg and Ebola viruses)
Cranial nerve palsy	Dysfunction of individual cranial nerves	**VZV**,** HSV**,** CMV**,** HIV**,** TBEV**, Mumps virus, Poliovirus, Hepatitis C virus
Ocular involvement	Chorioretinitis with visual impairment and potentially ocular pain	**CMV**,** HSV**,** VZV**
Slow-virus CNS infection	Personality changes, myoclonus, seizures, choreoathetoid movements, high mortality	**Measles virus**,** Rubella virus**

**Table 2:** Diagnostic criteria for the diagnosis of Encephalitis (based on the consensus paper of the International Encephalitis Consortium) [14]

Main Criteria (Primary Criterion)
▪ Decreased level of consciousness, personality changes, or psychiatric symptoms (lethargy) > 24 hours without an alternative cause
▪ No alternative diagnosis
**Additional Criteria**
▪ Fever (≥ 38 °C) within 72 hours before or after initial presentation
▪ Seizures not explainable by a pre-existing epilepsy
▪ New focal neurological signs
▪ WBC (CSF) ≥ 5/mm³
▪ Imaging abnormalities suggestive of encephalitis and appearing new or acute compared to prior imaging
▪ Abnormal EEG consistent with encephalitis and not explainable by another cause

### Epidemiology

At 10–20/100,000, the incidence of viral CNS infections in the USA is significantly higher than that of bacterial meningitis [15, 16]. In temperate climatic regions, the incidence of viral encephalitis is probably between 3.5 and 7.5 cases/100,000, with the highest incidence occurring in young and elderly people. Epidemics are excluded from this [17]. 

The most frequently identified viral pathogens in central nervous system (CNS) infections include enteroviruses (such as Coxsackie A, Coxsackie B, and echoviruses), followed by the mumps virus, arboviruses (including Flaviviruses, Bunyaviruses, and Alphaviruses), herpesviruses, human immunodeficiency virus (HIV), and lymphocytic choriomeningitis virus (LCMV). The epidemiological landscape of viral CNS infections has evolved over time. Widespread implementation of vaccination programs has led to a substantial reduction in the incidence of certain infections; for example, the introduction of the mumps vaccine has resulted in a marked decline in cases of mumps meningoencephalitis since the 1980s.

In contrast, an increasing number of Epstein-Barr virus (EBV) and cytomegalovirus (CMV) encephalitis cases are observed, particularly among immunocompromised individuals, such as those living with acquired immunodeficiency syndrome (AIDS), or undergoing organ transplantation or chemotherapy. Additional epidemiological factors influencing viral encephalitis include seasonality, geographic distribution, and exposure to vectors or reservoir animals. Consequently, the incidence and etiological spectrum of viral encephalitis vary significantly by region and context.

Arboviruses, including Zika virus, West Nile virus, and dengue virus, primarily cause viral infections during the summer months, when mosquitoes are most active. However, the activity of mosquitoes and ticks is not strictly confined to specific seasons. Studies have shown that Aedes aegypti and Aedes albopictus are capable of adapting to environmental conditions and ambient temperatures, enabling them to overwinter even in colder climates [18–20]. 

In North America, arboviral infections are of greater epidemiological significance than in Europe. Within Europe, infections caused by the tick-borne encephalitis (TBE) virus are particularly relevant. In Germany, TBE virus was responsible for 565 reported cases in 2022, 474 cases in 2023, and 268 cases by mid-July 2024. In Austria, 109 cases were documented in 2023, while Switzerland reported 245 cases in the same year.

For rubella virus-associated encephalitis, an estimated incidence of 1 in 24,000 cases has been reported [21]. Five rubella cases were documented in 2023, and ten cases were reported in 2024. Following the introduction of mandatory measles vaccination in 2020, the incidence of measles remained low, with 15 cases in 2022, 79 cases in 2023, and a notable increase to 632 cases in 2024. This recent rise may be attributable to inadequate vaccination coverage among refugee populations.

HSVE is the most common sporadic encephalitis in Western Europe with approx. 1–3 cases per 1 million. Individual cases have been observed after vaccinations (cholera, pertussis). Rabies (rabies) with its animal reservoirs of foxes, dogs and bats is considered to have been overcome in our country; worldwide, around 55,000 people still die of rabies every year. However, there are rabies viruses in native bats that can lead to infections and CNS diseases in humans. As the incubation period of the disease is very variable, there is a possibility that the disease may manifest itself in immigrants (depending on their region of origin) months after immigration.

The prevalence of viral manifestations in immunocompromised individuals has been reported as follows: herpes simplex virus (HSV) with necrotizing skin lesions—rarely progressing to encephalitis—in 4.0% of cases; varicella-zoster virus (VZV) complications, including herpes zoster and, less frequently, encephalitis, in 4.8%; progressive multifocal leukoencephalopathy (PML) in 1.8%; and cytomegalovirus (CMV) retinitis and encephalitis in 3.2% [22]. 

**Diagnostics**

- A viral infection should be considered as a potential etiology of an acute or subacute central nervous system (CNS) process, particularly in the presence of the following clinical history elements:
- Known exposure to viral infections in the patient’s environment (e.g., mumps, varicella, poliomyelitis).
- Tick bites (suggestive of tick-borne encephalitis [TBE]), insect bites (e.g., other arboviral diseases), or animal bites (e.g., rabies).
- Transfusion of blood products or organ transplantation (risk for HIV, cytomegalovirus [CMV], or parvovirus B19)
- Immunosuppression, whether disease-related or iatrogenic (risk for CMV, JC virus, varicella-zoster virus [VZV], human herpesvirus 6 [HHV-6], Epstein–Barr virus [EBV], and herpes simplex virus types 1 and 2). For instance, enterovirus 71 (EV-71) may cause CNS infections during anti-CD20 antibody therapy.
- Travel history, especially to regions endemic for viral encephalitides:
- Globally: rabies, dengue virus is on the rise.
- Italy: Toscana virus.
- Mediterranean region: West Nile virus.
- Southeast Asia: Japanese encephalitis virus, Nipah virus.
- North and Central America: West Nile virus, various alphavirus encephalitides.
- Central and West Africa: Lassa virus.
- Diagnosis relies on a combination of microbiological testing, clinical assessment, and neuroimaging. Electroencephalography (EEG) plays a crucial diagnostic role in identifying both subacute sclerosing panencephalitis (SSPE) and herpes simplex virus encephalitis (HSVE).
- Blood tests: A viral infection of the central nervous system (CNS) is suggested by the presence of relative lymphocytosis, even in the context of normal, slightly elevated, or reduced total leukocyte counts. In addition, procalcitonin levels typically remain below 0.5 ng/mL, whereas they are consistently elevated in acute bacterial CNS infections. Other blood parameters are usually within normal limits or non-specific. For example, C-reactive protein (CRP) may show a moderate elevation in acute viral CNS diseases, but values rarely exceed 50 mg/L [23, 24].

Serological diagnostic methods include ELISA for the detection of specific IgG, IgA, and IgM antibodies, immunoblot, immunofluorescence assays, and antibody avidity testing to estimate the time interval since acute infection. In addition, culture and virus isolation techniques may be employed where applicable.

- Cerebrospinal fluid (CSF) analysis during the first 4 to 48 h of a viral CNS infection may reveal a mixed pleocytosis ranging from 25 to 1000 cells/µL, consisting of lymphocytes, monocytes, and granulocytes. This typically evolves into a predominantly lymphocytic profile over time. Total protein and lactate levels are generally normal in viral meningitis, whereas in viral encephalitis, they may be mildly elevated, with lactate concentrations rarely exceeding 4.0 mmol/L.

An intrathecal synthesis of immunoglobulins is usually absent in viral meningitis and typically not yet detectable in the early phase of acute viral encephalitis. This immune response generally develops during the first weeks of illness (i.e., after > 10 days), particularly in encephalitis caused by herpes simplex virus (HSV), varicella-zoster virus (VZV), cytomegalovirus (CMV), and tick-borne encephalitis virus (TBEV).

A similar timeline applies to the intrathecal production of pathogen-specific antibodies, which can be measured using the antibody index (AI). In contrast, chronic viral encephalitides frequently show persistent intrathecal immunoglobulin synthesis, including the production of pathogen-specific antibodies with an AI > 1.5.

AI: (antibodies in CSF) x (serum IgG)/(CSF IgG) x (antibodies in serum).

Virological diagnostics allow for definitive pathogen identification in approximately 40% of patients with suspected encephalitis. In cases where no infectious agent can be detected, an autoimmune etiology—such as autoimmune encephalitis or acute disseminated encephalomyelitis (ADEM)—should be considered [25]. 

A 2016 consensus statement by Graus et al. provides a practical and widely adopted diagnostic framework for autoimmune encephalitis [3]. According to this guideline, a diagnosis of “possible autoimmune encephalitis” can be made if all three of the following criteria are met:

1. Short-term memory impairment, psychiatric symptoms or changes in.

consciousness with subacute onset (< 3 months),

(2) at least one of the following findings:

- new focal CNS symptoms.
- Cerebrospinal fluid pleocytosis (> 5 cells/mm^3^).
- epileptic seizures that cannot be explained by pre-existing epilepsy.
- MRI findings that indicate encephalitis.
  (3) justified exclusion of alternative causes.

Clinical laboratory assays [12, 26–29].

- Direct detection of viral DNA or RNA using PCR, multiplex PCR, or reverse transcription PCR (RT-PCR) from non-centrifuged cerebrospinal fluid (CSF). This method is applicable for a range of neurotropic viruses including HSV, VZV, CMV, EBV, JC polyomavirus, as well as flaviviruses and enteroviruses. In addition, cost-effective point-of-care tests are available for enterovirus detection.
- Detection of pathogen-specific IgM antibodies in CSF and/or serum using IgM ELISA, as is commonly done in West Nile virus (WNV) encephalitis.
- Furthermore, intrathecal production of pathogen-specific antibodies can be demonstrated by calculating the antibody index (AI).
- Conventional culture-based pathogen detection from body fluids, swabs, or biopsy-derived brain tissue is no longer considered relevant in routine clinical practice. However, it may still be used to detect enteroviruses and/or VZV in pediatric CSF samples when molecular diagnostics (e.g., PCR) are unavailable.
- In cases where the etiology remains unclear despite standard diagnostic workup, next-generation sequencing (NGS) of CSF may be employed to identify uncommon or unexpected pathogens.

### Neuroimaging

Magnetic resonance imaging (MRI) is the modality of choice for evaluating suspected viral encephalitis. Computed tomography (CT) should be considered only as an alternative—for example, in cases with contraindications to MRI or when there is a need to exclude complications (e.g., hemorrhage, mass effect) or critical differential diagnoses.

MRI plays a central role in both the differential diagnosis (e.g., distinguishing space-occupying lesions, abscesses, or autoimmune encephalitides) and in identifying disease-specific distribution patterns of inflammatory processes [30].

For instance, asymmetric basal ganglia involvement is characteristic of arboviral infections such as tick-borne encephalitis (TBE) and Japanese encephalitis virus (JEV), while temporal lobe lesions with frontobasal extension, seen as T2 hyperintensities and diffusion restriction, are typical of herpes simplex virus encephalitis.

### Electrophysiological Testing

Electroencephalography (EEG) can support the diagnosis of viral or autoimmune encephalopathies. Radermecker complexes are observed in prion-associated diseases, such as Creutzfeldt–Jakob disease (CJD), subacute sclerosing panencephalitis (SSPE), and progressive multifocal leukoencephalopathy (PML).

In HSVE, periodic lateralized epileptiform discharges (PLEDs) are considered pathognomonic. In anti-NMDA receptor encephalitis, the EEG may reveal the characteristic “extreme delta brush” pattern.

Table 3 shows the step-by-step diagnosis of neurotropic viruses in adults 

**Fig. 1:**
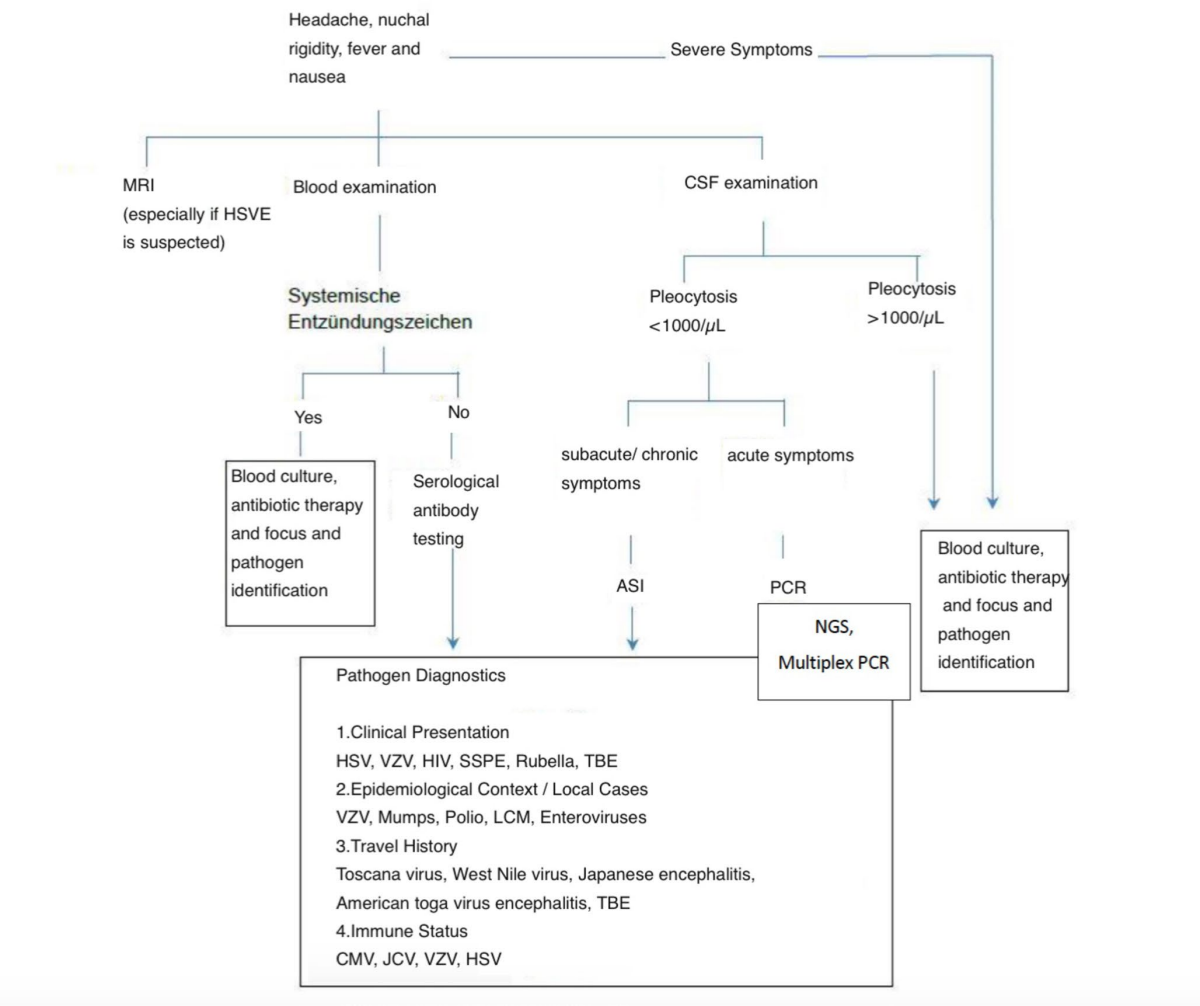
Diagnostic algorythm

Figure 1 shows the diagnostic algorithm as flow chart in case of suspected viral meningoencephalitis .

**Table 3:** Step-by-step diagnosis of neurotropic viruses in adults (modified according to) [13];

Stage 1: Specifically treatable viral infectionschoice diagnostic method	1. Choice diagnostic method	2.Choice diagnostic method
Herpes simplex virus 1/2 (HSV-1/−2)	DNA PCR	ASI (after 2 weeks)
Varicella zoster virus (VZV)	DNA PCR	ASI (after 2 weeks)
Cytomegalovirus (CMV)	DNA PCR	ASI, antigen (pp65) detection in cerebrospinal fluid and blood
Human immunodeficiency immunodeficiency virus 1/2 (HIV-1/2)	RNA-PCR, blood serology	ASI
**Level 2: Viruses for which potentially effective substances are known but not available**,** not sufficiently tested or not yet approved**		
Epstein-Barr virus (EBV)	DNA PCR	Special serology
Echoviruses, Coxsackieviruses	RNA-PCR, serology	Pathogen isolation
Nipah virus	RNA-PCR	Serology
**Level 3: more common viruses (Western Europe) that are not yet specifically treatable**		
Tick-borne encephalitis virus (TBE)	Serology in blood and cerebrospinal fluid (ASI)	RNA-PCR (early!)
Adenoviruses	Serology	Pathogen isolation
Human herpesvirus 6 (HHV-6)	Serology (ASI)	DNA PCR
Human herpesviruses 7, 8 (HHV-7/−8)	DNA PCR	Serology
Influenza virus A and B, parainfluenza	Serology	RNA-PCR
Measles virus	Serology (ASI)	RNA-PCR
Rubella virus	Serology (ASI)	RNA-PCR
JC virus (polyomavirus group)	DNA PCR	Serology
**Level 4: special constellations**		
***A) Special clinical syndromes***		
flaccid paresis: Poliovirus	Virus cultivation from cerebrospinal fluid and stool	RNA-PCR, serology
spastic paraparesis: HTLV-1	RNA-PCR, serology	
Suspected rabies: Rabies virus	RNA-PCR	direct immunofluorescence
Contact with rodents: LCM virus	Serology	PCR
Contact with excretions from mice and rats: Hantaviruses	Serology (ASI)	RNA-PCR
Hepatitis: Hepatitis C infection	RNA-PCR	Serology
***B) Stay abroad (see above; contact the Tropical Institute if necessary)***		

### Treatment

**General Therapeutic Principles**

In suspected encephalitis caused by herpes group viruses—particularly HSV or VZV—empirical antiviral therapy with aciclovir should be initiated without delay. For other viral encephalitides, treatment should be tailored to the identified pathogen based on current evidence and guideline recommendations.

If bacterial CNS infection cannot be definitively ruled out, empiric antibiotic therapy should be commenced (e.g., a third-generation cephalosporin combined with ampicillin; note the need to cover *Listeria monocytogenes* in cases of suspected listerial meningoencephalitis).

Passive immunization with hyperimmune sera is not indicated for tick-borne encephalitis (TBE) and should be reserved for rare pathogens, such as rabies virus, especially following confirmed or high-risk exposure or in epidemiologically justified scenarios involving severe viral infections.

The recommended general therapeutic measures are consistent across all severe cases of encephalitis:

Management of cerebral edema: Osmotherapy remains the primary approach. The therapeutic benefit of decompressive craniectomy has not been established.

Anticonvulsive therapy: Indicated only in the event of epileptic seizures or status epilepticus (refer to the DGN guideline “Status epilepticus in adults”).

Analgesics and sedatives: Administered as needed, based on symptom burden and clinical context.

Use of neuroleptics (e.g., haloperidol, melperone, olanzapine): Requires caution due to the potential lowering of the seizure threshold.

Intensive care management: Includes thrombosis prophylaxis and supportive treatment of autonomic dysregulation, disturbances in thermoregulation and respiration, cerebral salt-wasting syndrome, or diabetes insipidus.

Supportive care: Special attention should be paid to ensuring adequate nutritional support and strict temperature control.

For certain viral diseases with potential central nervous system (CNS) involvement—such as measles—prevention remains the primary strategy, particularly through active immunization.Early vaccination efforts have significantly reduced the incidence of both early and late complications associated with measles, rubella, mumps, and poliomyelitis [31]. 

In the case of sporadic or endemic viral infections such as tick-borne encephalitis (TBE) or rabies, targeted immunization of high-risk populations is recommended.

For TBE in particular, region-specific vaccination recommendations are available from the Standing Committee on Vaccination (STIKO) at the Robert Koch Institute [32].

1. Specialized therapies.

### Herpes simplex encephalitis (HSVE)

Herpes simplex virus encephalitis (HSVE) shows no clear seasonal pattern, in contrast to other viral encephalitides such as tick-borne encephalitis. The global annual incidence is estimated at roughly 0.2–0.4 cases per 100 000 population. In industrialized countries the incidence is lower, at about 1–3 cases per 1 000 000 population. Older studies from Western Europe report an incidence of approximately 5 cases per 1 000 000; however, this figure is likely an overestimate. Roughly one-third of HSVE cases occur in individuals younger than 20 years, whereas about one-half affect patients older than 50 years.

If left untreated, HSV encephalitis is fatal in at least 70% of cases. Individuals with recurrent herpes labialis are not at increased risk. In adults and older children, acute necrotising encephalitis is almost invariably caused by HSV type 1, whereas HSV type 2 in this age group more often results in a benign aseptic meningitis. In neonates, by contrast, HSV-2 typically produces a diffuse haemorrhagic-necrotising encephalitis rather than the focal lesions observed in adults.

Symptoms:

HSV-1 encephalitis is characterized by its biphasic course. An initial influenza-like prodrome—marked by headache and high fever—is frequently followed by a short interval of apparent improvement. Thereafter, patients develop qualitative or quantitative disturbances of consciousness accompanied by recurrent fever and headache, with focal neurological deficits occurring variably.

Ancillary diagnostics primarily serve to confirm the clinical suspicion of HSVE; antiviral therapy must begin immediately upon clinical suspicion and should not be delayed until test results (e.g., PCR) are available.

### CSF profile

Typically shows an initial mixed-cell pleocytosis that evolves within a few days into a lymphocytic pleocytosis of 5–350 cells/µL (higher counts are possible). About 5% of patients present with normal cell counts. A moderate-to-marked protein elevation and a mild lactate increase (≤ 4.0 mmol/L) are characteristic [33]. 

### Imaging

In MRI, early abnormalities appear medio-temporo-basally on DWI and FLAIR sequences; haemorrhage is a late finding. A normal MRI argues strongly against HSVE. Cranial CT usually becomes abnormal only ≥ 4 days after symptom onset and is therefore unsuitable for early diagnosis. Later findings include temporal and frontobasal hypodensities and involvement of the cingulate gyrus [34]. 

### EEG

Abnormal early, showing generalized slowing with periodic lateralized epileptiform discharges (PLEDs) that often correlate with focal clinical signs.

### Virological confirmation

CSF HSV PCR in the first days of illness: sensitivity 95–100% [35, 36].

From the end of week 2 onward, rising CSF antibody titres or evidence of intrathecal antibody synthesis confirm the diagnosis (sensitivity 97%, specificity 73–100%). PCR may become negative later in the course [37]. 

Up to 25% of patients experience a relapsing course, driven by viral reactivation or immune mechanisms. NMDA-receptor antibodies are increasingly detected in this context. In reported cases of NMDA-receptor-positive autoimmune encephalitis, escalation with corticosteroids and immunosuppressants such as rituximab or cyclophosphamide—in addition to anti-herpetic therapy—has been successful [38–40]. 

Early initiation of therapy can lower the case-fatality rate of HSVE to around 20%. Consequently, aciclovir should be started immediately on clinical suspicion, as delayed treatment initiation (> 24 h after admission) is linked to higher mortality [41]. 

Adjunctive dexamethasone has shown benefit in animal models, but the German Trial of Acyclovir and Corticosteroids in Herpes-Simplex Encephalitis (GACHE) was terminated early because of poor recruitment. Available data suggest that combined aciclovir–dexamethasone therapy does not worsen outcomes and may increase the proportion of patients achieving a very good outcome, although statistical significance was not reached owing to the small sample size. Thus, using dexamethasone alongside aciclovir remains an off-label, case-by-case decision [41]. 

Gnann et al. evaluated whether a 90-day oral valaciclovir maintenance course following initial intravenous aciclovir could lessen long-term cognitive sequelae. The trial demonstrated no outcome advantage for extended oral valaciclovir over standard therapy [42]. 

Intravenous aciclovir is to be administered at 10 mg/kg every eight hours for a minimum of 14 days; adequate hydration is to be maintained and the dose is to be reduced in cases of renal impairment. If CSF HSV-PCR is negative but clinical suspicion persists and no alternative cause is identified, aciclovir therapy should be continued for at least 10 days [12, 43]. 

In patients with AIDS or after organ transplantation, aciclovir-resistant HSV strains have been reported. In such cases, foscarnet should be given as an alternative at 60 mg/kg intravenously over 1 h every 8 h for 3 weeks.

### Varicella and Zoster encephalitis

Varicella-zoster virus (VZV) is the second most common cause of infectious encephalitis in industrialised countries, with neurological complications reported in approximately 55% of cases. Central nervous system involvement occurs in ~ 0.1% of primary varicella (chickenpox) infections and typically manifests 4–8 days after the onset of the rash. In about half of these patients, cerebellar signs predominate; otherwise, cerebral or spinal symptoms are foremost [17, 58].Vasculitis associated with VZV is a rare cause of stroke, Kraemer et al. [59] published a small case series. They described three young men with varicella‑zoster–associated intracranial vasculitis whose severe arterial stenoses improved markedly—two almost completely—after many months of combined valacyclovir and prednisolone therapy, suggesting that even pronounced vessel narrowing can be reversible with prolonged antiviral and anti‑inflammatory treatment, though steroid‑related side effects and the very limited evidence base highlight the need for careful clinical judgment and further research [59]. 

Treatment with intravenous aciclovir with doses identical to HSVEis recommended, although large randomised trials are lacking [44]. Brivudine may be used as an alternative; however, concomitant administration of fluorouracil or related fluoropyrimidines is contraindicated. According to current safety data, the use of 5-fluorouracil, capecitabine, tegafur, or flucytosine shortly before, during, or within four weeks after brivudine therapy carries a potentially fatal risk of toxicity.

The following preparations are available for the treatment of uncomplicated zoster: Acyclovir (5 times 800 mg orally for 7–10 days), famciclovir (3 times 250–500 mg orally) and brivudine (125 mg/d; start therapy within the first 72 h after the appearance of efflorescences for 7 days).

Zoster ophthalmicus is treated with i.v. aciclovir (see Table 4).

Zoster encephalitis—either as a complication of cutaneous zoster or as a primary encephalitic presentation—predominantly affects individuals with leukaemia, lymphoma, or other forms of immunodeficiency. Central-nervous-system manifestations typically emerge a few days to weeks after the appearance of vesicular skin lesions, which are most often confined to cranial dermatomes.

Intravenous aciclovir is the treatment of choice for zoster encephalitis (see Table 4). Although the clinical course may resemble that of HSV-1 encephalitis, progression is typically slower and the disease is usually less severe; nonetheless, residual deficits and fatal outcomes are not uncommon. Early initiation of aciclovir is critical for a favourable prognosis. If aciclovir proves insufficient, zoster encephalitis may be treated with foscarnet as an alternative (dose as for CMV infections; see Table 4).

**Table 4:** Available antiviral substances with probable or confirmed efficacy in CNS infections [45]

Antiviral Substance	Proven Efficacy	Possible Efficacy	Pharmacological Properties
Aciclovir/Valaciclovir	Herpes simplex, Varicella-zoster, Herpes simiae virus	Epstein-Barr virus	Oral bioavailability: 10–20%, Plasma half-life: 2–3 h; Intracellular half-life: 1–2 h
Penciclovir/Famciclovir	Herpes simplex, Varicella-zoster virus	Herpes simplex encephalitis (no studies), Hepatitis B	Oral bioavailability: 77%, Plasma half-life: 2 h; Intracellular half-life: 7–20 h
Ganciclovir/Valganciclovir	Cytomegalovirus (CMV)	HSV, VZV, EBV, HHV 8, Herpes simiae virus	Oral bioavailability: 8–9%, Plasma half-life: 2.5 h; Intracellular half-life: 12 h
Foscarnet	CMV, Aciclovir-resistant VZV and HSV	HHV 8, HIV 1	Oral bioavailability: 0% (i.v. only!), Plasma half-life: 6 h; Triphasic elimination due to deposition in bone matrix
Ribavirin	Hantaviruses (hemorrhagic fever), Hepatitis C (in combination with interferon-α)	Hantavirus (pulmonary syndrome), Measles, Parainfluenza, Influenza A and B	Oral bioavailability: 32%, Plasma half-life: 32 h; also available as aerosol
Interferon-α	Hepatitis B and C, HHV 8		Oral bioavailability: 0% (i.v. only!), Plasma half-life: 3–8 h
Cidofovir (with Probenecid)	CMV retinitis*	HSV, VZV, EBV, JC virus**	Oral bioavailability: 0%, Plasma half-life: 3 h; Intracellular half-life: 24–65 h

* [46]

** [47]

### Cytomegalovirus (CMV) Infections

CMV can cause severe encephalitis and permanent deficit syndromes when infection occurs prenatally or perinatally. In childhood, adolescence, and adulthood, primary CMV infection is usually asymptomatic; when illness does occur, it typically resembles infectious mononucleosis. Acute or chronic CMV involvement of the nervous system is seen almost exclusively in immunocompromised patients. In AIDS, CMV presents mainly as encephalitis and/or chorioretinitis, and cerebrospinal fluid (CSF) may show a granulocytic pleocytosis.

Further recommendations for the diagnosis and management of CMV disease with CNS involvement have been issued by the International Herpes Management Forum (IHMF): [48]

### Diagnosis and Treatment

CSF PCR for CMV DNA is mandatory to confirm the diagnosis.

### Initial phase (3 weeks)

Ganciclovir 5 mg/kg i.v. every 12 h plus.

Foscarnet 60 mg/kg i.v. every 8 h or 90 mg/kg i.v. every 12 h.

Combination therapy is recommended because ganciclovir alone has limited efficacy against CMV encephalitis.

### Consolidation/maintenance

Ganciclovir monotherapy is continued for.

3 weeks in immunocompetent patients.

6 weeks in immunocompromised patients.

If oral therapy is feasible, valganciclovir may be substituted (900 mg p.o. twice daily for 3 weeks, then 900 mg once daily). Valganciclovir is proven effective for CMV retinitis, although data for CMV encephalitis are lacking [49]. 

### Second-line agents

Cidofovir 5 mg/kg i.v. once weekly, given with probenecid (2 g 3 h before and 2 h/8 h after infusion), can be used when ganciclovir or foscarnet are ineffective or resistance is suspected. Both foscarnet and cidofovir are more toxic than ganciclovir, and cidofovir is considered potentially carcinogenic and mutagenic [48, 50]. 

### Prognosis and Follow-up

Therapeutic response is generally favourable in CMV chorioretinitis, whereas outcomes for other CNS manifestations remain uncertain. In AIDS patients, maintenance therapy is necessary after acute treatment to prevent relapse.

### Epstein-Barr virus encephalitis

Epstein–Barr virus (EBV) encephalitis occurs predominantly in immunosuppressed individuals, such as organ transplant recipients. Typical clinical features include fever, confusion, nausea, vomiting, and altered consciousness, with focal neurological deficits present in some cases. EEG findings are nonspecific. Diagnostic confirmation relies on detection of EBV DNA in cerebrospinal fluid (PCR), bearing in mind that pleocytosis may yield false-positive results; intrathecal antibody synthesis is unreliable in immunocompromised patients. Therapeutic attempts—both in immunocompetent and immunosuppressed hosts—have largely consisted of aciclovir or valaciclovir, usually combined with high-dose corticosteroids [51–53]. 

### Progressive multifocal leukoencephalopathy (PML)

PML is caused by the JC virus (JCV), a non-enveloped DNA polyomavirus that primarily infects oligodendrocytes but can also involve neurons and cerebellar granule cells.

- Adult exposure rate: ~ 92%.
- Age-dependent seroprevalence: ~ 30–7%, increasing with age.
- Clinically manifest PML occurs almost exclusively in individuals with immune defects, malignancies, or iatrogenic immunosuppression. ≈ 85% of cases are HIV-associated.
- Cases have also been linked to immunomodulatory therapies such as natalizumab, rituximab, and mitoxantrone. Plasma exchange has occasionally improved outcomes in natalizumab-associated PML.

### Clinical Presentation

Early signs often include neuropsychological changes and visual disturbances. Additional features may be paresis, aphasia, seizures, ataxia, dysarthria, headache, or nonspecific dizziness. Late-stage disease is marked by dementia, ataxia, tetraparesis, cortical blindness, and preterminal decerebration.

### Diagnosis

#### Clinical context

HIV infection, immunosuppression, lymphoproliferative disease, or monoclonal-antibody therapy.

#### MRI

Large, asymmetric T2 hyperintensities—typically parieto-occipital—with juxtacortical white-matter involvement; “milky-way” punctate lesions may appear at lesion margins. Contrast enhancement is usually absent (except in PML-IRIS).

#### Laboratory

JCV DNA in CSF by PCR confirms the diagnosis, though sensitivity may be lowered by viral hypermutation; repeat testing in reference labs is advised when suspicion remains high. Brain biopsy is reserved for select PCR-negative cases.

#### Biomarker

Ultrasensitive neurofilament light chain (NfL) assays may help distinguish active PML lesions.

### Treatment Options

#### Prognosis

Natalizumab-associated PML has a better outlook than HIV-associated disease (estimated mortality 20–5%) owing to reversible immunosuppression. In most other settings, PML remains frequently fatal.

#### Mollaret meningitis

Mollaret’s meningitis represents a distinct form of benign, chronically recurrent aseptic meningitis. Herpes simplex virus—most often HSV-2—is presumed to be the principal aetiological agent. Cerebrospinal fluid typically shows pleocytosis containing large endothelial “Mollaret cells,” which are characteristic but not pathognomonic; HSV-2 DNA can occasionally be detected by PCR. The disease is usually self-limiting, although episodes may recur over several years. Important differential diagnoses include drug-induced aseptic meningitis (DIAM), which presents with identical clinical features and is most commonly triggered by NSAIDs (e.g., ibuprofen) or certain antibiotics [54, 55]. 

#### Chronic encephalitis

The The two encephalitides with a proven “slow-virus” pathogenesis are subacute sclerosing panencephalitis (SSPE) and progressive rubella panencephalitis (PRP).

Both conditions are characterized by exceptionally long incubation periods—ranging from months to years—and a protracted, chronically progressive course that invariably leads to death.

#### Epidemiology

These conditions occur almost exclusively in childhood and adolescence. The incidence of SSPE is approximately 1 in 1 million per year in the general population, but significantly higher—around 1 in 3,300—in children under five years, with a predominance in males. Since the introduction of measles vaccination, SSPE has become rare; however, reported measles cases in Germany have fluctuated, with 15 cases in 2022, 79 in 2023, and already 632 cases reported in 2024. Most affected children were either unvaccinated or vaccinated too late, often belonging to socially disadvantaged or migrant families [56]. 

Since implementation of measles vaccination, SSPE has become rare, yet notified measles cases in Germany have fluctuated: 15 in 2022, 79 in 2023, and 632 already reported in 2024 (Robert Koch Institute). Most affected children were unvaccinated or vaccinated too late, often from socially disadvantaged or migrant families.

#### Clinical course

Both disorders are marked by behavioural changes, personality deterioration, and dementia; myoclonic jerks are characteristic of SSPE. Ultimately, both conditions progress to a coma vigile before resulting in death.

#### Diagnosis

In SSPE, the diagnosis is supported by excessive intrathecal synthesis of measles antibodies, indicated by an antibody index greater than 1.5, and/or the detection of SSPE antigen. The EEG typically reveals the almost pathognomonic Radermecker complexes. In PRP, diagnosis is similarly confirmed through the detection of intrathecal rubella antibodies.

#### Management and prognosis

There is no curative therapy for either SSPE or PRP; both conditions are essentially untreatable and invariably fatal. β-Interferon has shown some potential to slow disease progression in isolated cases, although the evidence remains inconsistent. Early measles vaccination continues to be the only effective preventive measure against SSPE.

## Rabies

Rabies, caused by Lyssaviruses and predominantly transmitted by dogs, remains a significant cause of infectious disease-related mortality. The World Health Organization (WHO) estimates approximately 60,000 annual deaths worldwide, with around 95% of these cases occurring in Asia and Africa. Cases in Germany are extremely rare due to widespread vaccination efforts, and Germany, like most European countries, is considered rabies-free. Nevertheless, native bats in Germany can carry rabies viruses, potentially causing human infections and illness. Infection risks in Germany primarily result from travel to countries where rabies is endemic.

Clinically, rabies symptoms can be categorized into a prodromal phase and an acute neurological phase. The prodromal stage presents nonspecifically, with symptoms such as fever, headache, general malaise, and itching or burning sensations at the bite wound site. The acute neurological phase manifests as either encephalitic rabies (approximately 80% of cases) or paralytic rabies (around 20%). When obtaining travel history, it is crucial to consider that rabies incubation periods can range from three weeks to three months, and exceptionally, up to several years.

The last rabies case reported to the Robert Koch Institute occurred in 2007, and the most recent suspected rabies exposure was reported in 2015. According to the latest surveillance report from the European Centre for Disease Prevention and Control (ECDC), covering data from 2019, five cases of human lyssavirus infections were recorded in the EU. One autochthonous case from France was caused by a bat-transmitted European bat lyssavirus. Four travel-associated cases were reported from Italy, Lithuania, Spain, and Norway, with travelers returning from Tanzania, India, Morocco, and the Philippines.

## Emerging Viruses

### Monkeypox

Human monkeypox virus (hMPXV) is an Orthopoxvirus closely related to variola, the agent of classical smallpox. Although the clinical picture resembles smallpox, hMPXV infection is generally milder. Transmission occurs via direct contact with skin, mucous membranes, or lesion material—most often during sexual activity. Both vesicular fluid and scabs are highly infectious, harbouring large viral loads; lesions may be clinically occult.

Incubation: 5–21 days (shorter intervals have been reported during recent outbreaks).

**Clinical course**

1. Prodromal stage: fever, malaise, headache, myalgia, and/or lymphadenopathy.
2. Exanthem stage: painful or pruritic maculopapular rash that progresses to vesiculopustular lesions of uniform size, sharply demarcated and occasionally umbilicated, distributed over the face, oral mucosa, trunk, and extremities—including palms and soles.

Illness usually lasts 2–4 weeks with low mortality in immunocompetent individuals.

### Neurological involvement

Neurological manifestations are uncommon. A recent meta-analysis suggests headache is the most frequent neurological symptom [57]. WHO reports headache in 31% of 37 130 global cases. hMPXV-associated encephalitis should be considered when compatible neurological signs coincide with vesiculopustular lesions.

### Management

Treatment is supportive; prognosis is favourable in most cases.

#### Ebola virus Disease (EVD)

The onset of an EVD is quite abrupt. At the end of December 2013, the Ebola outbreak in West Africa had claimed 8371 lives, with a total of 21,171, including 13,397 laboratory-confirmed cases of the disease. The last Ebola outbreak in 2022/2023 was caused by the Sudanese Ebola virus. From 20.9.2022 to 10.1.2023, over 160 people were infected in the western and central regions of Uganda, resulting in 77 deaths.

Ebola viruses were first discovered in 1976 when there were two consecutive outbreaks of deadly hemorrhagic fever in different parts of Central Africa.

Initially, health authorities assumed that these outbreaks were a single event linked to an infected person traveling between the two locations. Scientists later discovered that the two outbreaks were caused by two genetically different viruses: the Zaire Ebola virus and the Sudan Ebola virus. It is believed that African fruit bats are probably involved in the spread of Ebola viruses and may even be their reservoir hosts. As with other viruses of this type, it is possible that the reservoir host animal does not suffer serious illness despite being infected with the virus. Ebola viruses are likely to persist in the environment by spreading from host to host or via intermediate hosts or vectors.

The clinical symptoms range from fever, headache and muscle pain to encephalitis. With a lethality rate of 70–90%, multifocal nodular encephalitis with perivascular hemorrhages develops. Death usually after 6 to 9 days. Various methods are available forlaboratory diagnosis: PCR, virus isolation serological detection using ELISA, whereby diagnostics in Germany may only be out in certain laboratories. The shipment of samples is also subject to strict guidelines.

There are currently two drugs approved by the US Food and Drug Administration (FDA) for the treatment of EVD caused by the Ebola virus, species Zaire Ebola virus, in adults and children. Inmazeb™ is a combination of three monoclonal antibodies. The second drug, Ebanga™, is a single monoclonal antibody.

#### Dengue virus (DENV)

Dengue fever is among the most common infectious diseases with pandemic proportions, with its overall incidence increasing more than 30-fold over the past 50 years. Estimates suggest approximately 390 million cases occur annually worldwide. Since World War II, dengue fever has been classified as an emerging disease, with cases having increased 30-fold from 1960 to 2010. According to the World Health Organization (WHO), half of the world’s population lives in endemic areas, and at least 20,000 to 30,000 deaths occur annually.

Dengue virus (DENV) is an arbovirus with four known serotypes (DENV 1–4), primarily transmitted by mosquitoes of the genera Aedes aegypti and Aedes albopictus. After an incubation period of 3–14 days, most DENV infections are mild and oligosymptomatic. Typically, symptoms include a sudden fever, severe headache, muscle and joint pain, bradycardia, hypotension, and lymphadenopathy. Occasionally, a second peak of fever may occur, accompanied by a generalized rash, petechiae, and thrombocytopenia. The classic dengue triad consists of fever, rash, and significant head, muscle, and joint pain. The disease course is generally short and abrupt, although prolonged convalescence with severe fatigue lasting several weeks can also occur.

Thrombocytopenia and platelet dysfunction commonly occur, and hepatitis with moderate elevation of transaminases is frequent. Approximately 10% of cases develop encephalitis, often followed by marked fatigue lasting several weeks post-acute illness, indicative of chronic fatigue syndrome. Severe forms of dengue include dengue hemorrhagic fever (DHF) and dengue shock syndrome (DSS), often occurring as severe secondary infections.

Laboratory diagnosis is conducted from cerebrospinal fluid or serum samples. There is no specific antiviral therapy available, and management is symptomatic. Although a vaccine exists for endemic areas, the World Health Organization currently provides no general recommendation for its use.

#### Zika virus (ZIKV)

The Zika virus (ZIKV) was first isolated in 1947 from a rhesus monkey in the Zika Forest, Uganda, Africa. Transmission primarily occurs through mosquitoes, most notably Aedes africanus. ZIKV belongs to the arboviruses, similar to dengue virus (DENV). Recent scientific field studies have demonstrated that mosquitoes can simultaneously transmit multiple viruses to humans, known as cross-infections.

ZIKV infection may present with symptoms including fever, rash, conjunctivitis, muscle and joint pain, nausea, or headaches. Generally, symptoms associated with ZIKV infections are mild. Neurological complications such as Guillain-Barré syndrome-like presentations, neuropathies, and myelitis have been described.

Congenital syndromes can be triggered by ZIKV during pregnancy. A causal relationship between ZIKV infection and malformations such as microcephaly and other severe brain abnormalities has now been established. These malformations result from the virus crossing the placenta and infecting fetal neural cells.

Laboratory diagnosis is performed using cerebrospinal fluid or serum samples. Currently, there is no specific antiviral treatment available, and management remains exclusively symptomatic. Travelers to ZIKV-endemic regions are advised to ensure adequate insect protection. Despite precautions, the Robert Koch Institute (RKI) continues to document increasing numbers of cases among returning travelers in Germany. As of 2024, 30 cases have been reported. Due to the COVID-19 pandemic and associated travel restrictions, case numbers had declined in recent years (2019: 11 cases; 2020: 6 cases; 2021: 2 cases). In 2023, cases began returning to previous levels with 16 cases reported, and numbers are currently on a slight upward trend.

## Data Availability

All included data´s are listed in the literature, there are no data generated for this publication.

## Abbreviations

AI: Antibody index
ATM: Acute transverse myelitis
CJD: Creutzfeldt–Jakob disease
CMV: Cytomegalyvirus
CNS: Central nervous system
COVID- 19: coronavirus disease 2019 / SARS-CoV-2
CSF: Cerebrospinal fluid
CT: Computed tomography
DENV: Dengue virus
DHF: Dengue hemorrhagic fever
DIAM: drug-induced aseptic meningitis
DSS: Dengue shock syndrome
ECDC: European Centre for Disease Prevention and Control
EBV: Epstein-Barr virus
EEG: Electroencephalography
EVD: Ebola virus disease
FDA: US Food and Drug Administration
HIV: Human immunodeficiency virus
HSVE: Herpes-simplex virus encephalitis
HSV-1: Herpes virus type 1
HSV-2: Herpes virus Type 2
hMPXV: Human monkey pox virus
IHMF: International Herpes Management Forum
JEV: Japanese encephalitis virus
LCMV: lymphocytic choriomeningitis virus
MRI: Magnetic resonance image
NfL: neurofilament light chain
NGS: New genome sequencing
PCR: Polymerase chain reaction
PLEDs: periodic lateralized epileptiform discharges
PML: Progressive multifocal leukoencephalopathy
PRP: progressive rubella panencephalitis
RKI: Robert Koch Institute
SSPE: subacute sclerosing panencephalitis
STIKO: Standing Committee on Vaccination
TBE: Tick borne encephalitis
VZV: Varicella-zoster virus
WHO: World Health Organization
WNV: West Nile virus
ZIKV: Zika virus

## Declarations

### Methological approach

The project was managed by the coordinator Prof. Dr. Uta Meyding-Lamadé. The topics were worked on by author-teams based on the current data situation and then coordinated in a first Delphi round by the guideline group.

This S1 level guideline (AWMF-registry number 030–100) is based on a systematic pubmed search. Where possible, the following sources were used: pubmed.gov; springer search; google scholar.

The core statements were evaluated according to the guidelines of the Oxford Centre for Evidence-based Medicine - Levels of Evidence and from this a strength of recommendation was derived. A strong recommendation corresponds in the formulation to a “should”, a recommendation to a “should” and an open recommendation to a “can”.

In a second Delphi round, all recommendations were finally agreed upon by the neurological guidelines group. Based on this expert consensus, the formulation of the core statements was evaluated as strong agreement in the case of > 95% of all experts, as agreement in the case of 75–95%, as majority agreement in the case of > 50–75%, and as no agreement in the case of < 50%. In this abbreviated guideline we only refer to agreements of 90–100%.

The guideline was reviewed by the Guideline Committee of the German Neurological Society and approved by the German Neurological Society. Interdisciplinarity was established.

Patient organizations were not involved.
